# Heterogeneous Electron‐Transfer Rates for the Reduction of Viologen Derivatives at Platinum and Bismuth Electrodes in Acetonitrile

**DOI:** 10.1002/celc.201600536

**Published:** 2016-11-25

**Authors:** Shaun K. Cook, Benjamin R. Horrocks

**Affiliations:** ^1^ Chemical Nanoscience LaboratorySchool of Chemistry, Bedson BuildingNewcastle UniversityNewcastle upon TyneNE1 7RUUK

**Keywords:** bismuth, electrochemistry, electron transfer, redox chemistry, surface chemistry

## Abstract

The standard heterogeneous rate constants for the reduction of a series of viologen derivatives with a range of inter‐ring torsion angles were measured at Bi and Pt electrodes. The electrode potentials for the first one‐electron reduction of the viologens vary from −684 mV to −1070 mV vs. Ag/0.01 m Ag^+^; this enabled a comparison of the behaviour of metallic (Pt) and semi‐metallic (Bi) electrodes over a wide range of applied potentials. The differential capacitance (6.5 μF cm^−2^) of Bi/MeCN,TBAPF_6_ interfaces at the potential of zero charge (*pzc*=−0.60 V) is at least an order of magnitude greater than that calculated on the basis of the bulk Bi carrier density (3×10^17^ cm^−3^) and the differential capacitance (9.5 μF cm^−2^) of Pt/MeCN interfaces at their *pzc* (−0.43 V) is of the same order. The series of viologen derivatives exhibited simple one‐electron redox behaviour and showed similar rate constants at Pt (1.8×10^−4^–1.6×10^−3^ cm s^−1^) and Bi electrodes (1.1×10^−4^–1.9×10^−3^ cm s^−1^) after application of the Frumkin correction. These results demonstrate that the density of states at the Bi surface is much higher than in bulk. Finally, the Frumkin‐corrected standard rate constants were observed to be inversely correlated with the inter‐ring torsion angle of the viologens.

## 1 Introduction

In general, the rate of heterogeneous electron transfer (ET) depends on both the nature of the redox couple and the electrode material. Typical electrode materials (Au, Pt) have densities of states (DOS) in a relatively narrow range 10^22^–10^23^ cm^−3^ eV^−1^; the effect of the electrode material on the rate is therefore difficult to study. This is important because a DOS‐independent ET rate is a signature of an adiabatic process.^1^, ^2^, ^3^, ^4^

The most common electrode material with a low DOS at the Fermi level is graphite, a semi‐metal. ET rates at highly oriented pyrolytic graphite (HOPG) have been extensively studied, but rather different conclusions have been reached by different authors. Low basal‐plane ET rates have been observed by some authors^5^, ^6^, ^7^, ^8^, ^9^, ^10^, ^11^ and much higher rates, comparable to those at noble metals by others.^12^, ^13^, ^14^ HOPG is a layered material and higher ET rates have been observed at step edges.^15^, ^16^, ^17^, ^18^ Enhanced rates at step edges on the layered semiconductor WSe_2_ were also observed by scanning electrochemical microscopy.^19^ Increasingly, sophisticated approaches based on combined electrochemistry‐microscopy and variants of scanning electrochemical microscopy, which allow localised measurement of ET rates, have been brought to bear on the problem and another interpretation of the step edge effect in terms of delamination of the layers has been suggested.^14^ The area has been reviewed recently.^20^, ^21^

Bismuth is a semi‐metal with a low density of states in the bulk, but unlike HPG does not have a layered structure. Bi electrodes have found use as alternatives to mercury electrodes because of their relative non‐toxicity and the ease with which smooth Bi surfaces can be prepared.^22^, ^23^, ^24^, ^25^, ^26^, ^27^ The use of Bi in electrochemistry is much less common than the use of noble metals, mainly because Bi is more susceptible to oxidation. However, within the potential window, voltammetric experiments performed at a polycrystalline Bi electrode can yield results of similar quality to those obtained at noble metal surfaces and it remains an interesting and useful electrode material, especially for cathodic reactions.^28^, ^29^

**Figure 1:**
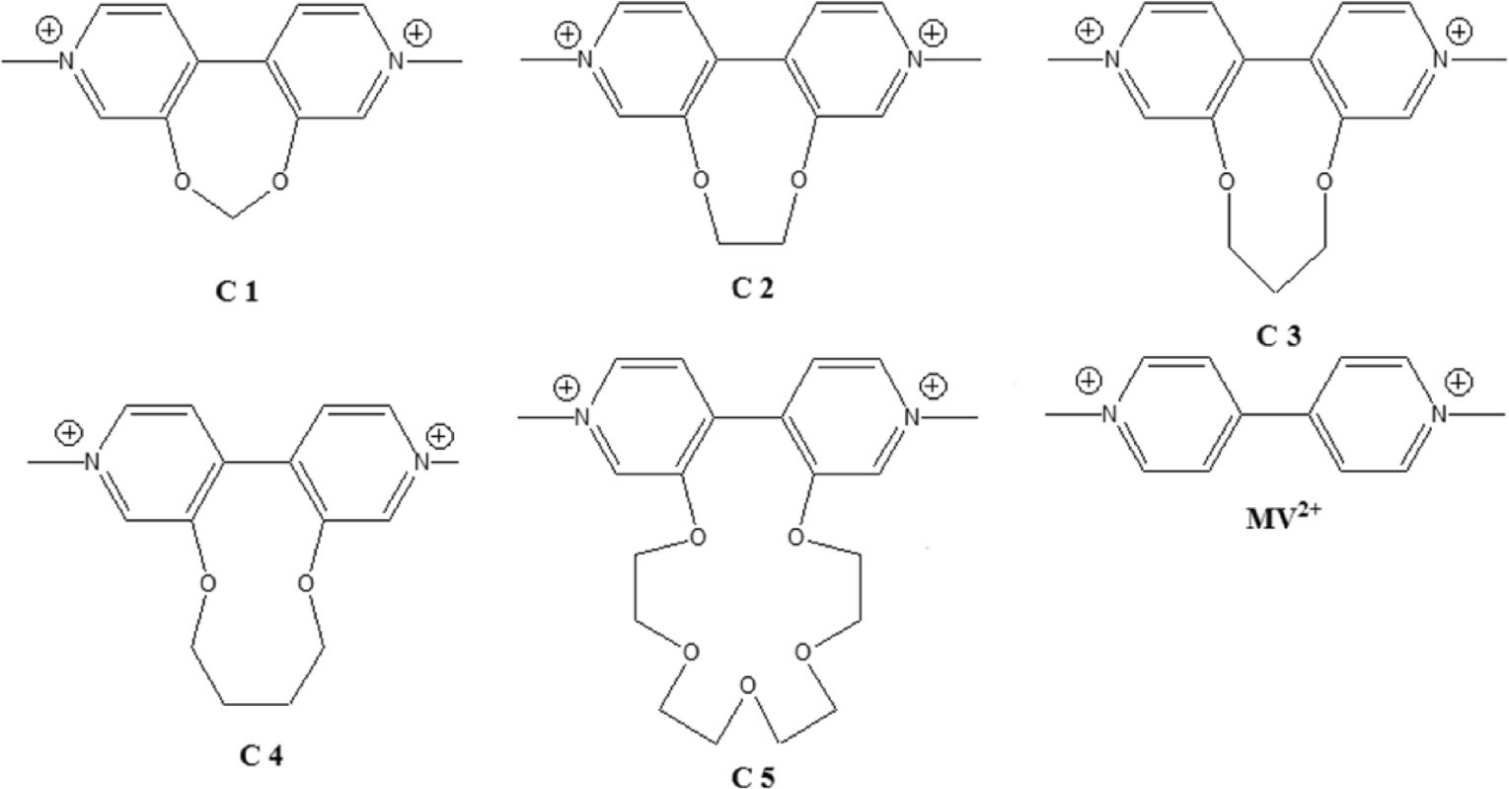
Methyl viologen (MV^2+^) and the series of derivatives studied in this work (**C1**–**C5**).

In this report we investigate the electrode kinetics of a series of viologen derivatives which undergo simple one‐electron reductions at both Pt and Bi electrodes. These viologen derivatives possess a linking tether that constrains the torsion angle between the two pyridyl rings (**C1**–**C5**, Figure 1).^30^ This series of molecules is well suited for investigations of heterogeneous electron‐transfer rate constants because each molecule in the series has similar electrochemical behaviour and they span a range of electrode potentials within the operational range of the Bi electrode. In particular, the inter‐ring torsion angle is controlled by the tether and allows us to examine the effect of this factor on the rate of the one‐electron reduction in addition to the difference between Pt and Bi.

Methyl viologen (MV^2+^, Figure 1) belongs to a class of 1,1′‐disubstituted‐4,4′‐bipyridinium salts, which is a group of dications capable of being reversibly reduced to stable radical cations or neutral species, collectively referred to as the viologens. These molecules have long been known,^31^ and they are used as herbicides.^32^ More recently, interest has surrounded their use in electrochromic systems,^33^, ^34^, ^35^, ^36^ in artificial photosynthesis,^37^ and in chemical sensors.^38^, ^39^ The viologens are well suited to the study of electron‐transfer phenomena because of their ability to form a stable radical following a one‐electron reduction, and to be subsequently reoxidised to reform the dication.^31^ Though air sensitive,^40^ it has been shown that the methyl viologen radical cation may be isolated from non‐aqueous solutions.^41^ Although there is little barrier to rotation, the lowest energy conformation in solution has a torsional angle between the two phenyl rings near 45°. Computational studies predict that a large decrease in the torsional angle in the lowest energy conformer occurs following reduction.^30^, ^42^ We therefore expect that by constraining this torsional angle, the rate of the electron transfer reaction will be modified because of the change in the inner sphere reorganisation energy of the molecule with an otherwise minimal impact on redox behaviour. We have looked for this effect in our series of tethered methyl viologen derivatives, which exhibit a range of inter‐ring torsional angles.

To our knowledge, the standard electron‐transfer rate constant for the one‐electron reduction of methyl viologen in acetonitrile has never been reported. Attempts to study the rate in aqueous solvents have been made but they are complicated by adsorption phenomena and dimerisation of the reaction products.^43^, ^44^, ^45^ Electrode fouling due to electrodeposition of the neutral form of the molecule has also been observed in aqueous solvents.^46^ We show that in acetonitrile, repeatable measurements of the standard rate constant may be obtained, free from such complications, by using fast scan voltammetry and convolution analysis.^47^ First we present the results of slow‐scan cyclic voltammetry experiments and demonstrate that the one‐electron reduction of the viologen derivatives is a well‐behaved, simple electron transfer, then we discuss the measurements of standard electrochemical rate constants by using fast‐scan cyclic voltammetry (FSV), impedance spectroscopy (EIS) and steady‐state microelectrode voltammetry. The differential capacitance of the Pt and Bi electrodes and ionic strength effects on the steady‐state voltammetry are used to determine aspects of the double‐layer structure necessary to make the Frumkin correction of the raw standard rate constants. Finally, we compare the electron‐transfer rates at Pt and Bi to theoretical predictions and discuss the correlations of molecular structural features with the rate constants.

## Experimental Section

Tetrabutylamonium hexafluorophosphate (TBAPF_6_) was purchased from Sigma–Aldrich and used as received. The PF_6_^−^ salt of MV^2+^ was prepared by the metathesis reaction of aqueous methyl viologen dichloride hydrate with NH_4_PF_6_ and washed with deionised water; both reagents were obtained from Sigma–Aldrich. Deionised water was obtained from a Barnstead Nanopure^TM^ purification train with nominal 18.2 MΩ cm resistivity. ReagentPlus^TM^ Acetonitrile (MeCN) was purchased from Sigma–Aldrich, dried over calcium hydride and distilled prior to use. The electrochemical cell used was deoxygenated before each experiment by sparging with nitrogen for 15 min. All potentials were measured against a Ag/Ag^+^ (10 mm in MeCN) reference electrode (IJCambria Ltd, UK) and a tungsten wire was used as the counter electrode. A Pt working electrode of radius 1 mm was used for slow‐scan cyclic voltammetry, and Pt microelectrodes of 25 μm and 5 μm radius were used in fast‐scan cyclic voltammetry (FSV), impedance spectroscopy (EIS) and steady‐state voltammetric experiments. Microelectrodes of 25 μm radius were prepared by sealing Pt wire into a glass tube, cutting through the glass to reveal a Pt disc, and then polishing this disc to give a flat surface. The 5 μm electrodes were SECM tips purchased from IJ Cambria Ltd. (UK). Pt wire was purchased from Goodfellow (Cambridge, UK), as were Pt foil substrates for use in scanning electrochemical microscopy (SECM). Bi powder (99.99 %) was obtained from Sigma–Aldrich. Bi disc electrodes were created by melting this powder in a thick‐walled soft glass tube of 1 mm bore. The tube was heated with a glassblower's torch and a copper wire was inserted into the molten Bi as contact. After cooling and solidification, the end was polished flat by using diamond paste on a nylon pad with a polishing wheel, taking care not to expose the copper wire at the surface of the disc. The solutions studied contained TBAPF_6_ electrolyte at a typical concentration of 0.1 m or 0.2 m in MeCN, and the methyl viologen derivative at a concentration of 1 mm or 10 mm. All electrochemical measurements were performed with a CH Instruments 760b potentiostat except the steady‐state microelectrode measurements, which were performed with a CH Instruments 900 scanning electrochemical microscope, and differential capacitance measurements, which were performed with an Ivium CompactStat e (Alvatek, UK).

The methyl viologen derivatives used in this study were a gift from Profs. A. Harriman and A. C. Benniston (School of Chemistry, Newcastle University) and were prepared and purified as has been described previously.^30^

## 2 Results and Discussion

### 2.1 Characterisation of the Redox Reactions of Methylviologen Derivatives

As preparation for the kinetic measurements, a series of standard voltammetric experiments were conducted to characterise the electrochemistry of the viologen/acetonitrile system. Formal potentials, adsorption and fouling effects were investigated by using cyclic voltammetry.

#### 2.1.1 Slow‐Scan Cyclic Voltammetry

Formal potentials for the first and second reductions of MV^2+^ and **C1**–**C5** were measured by cyclic voltammetry and are collected in Table 1. The peak separations were all close to 60 mV for the first reduction and the compounds showed reversible behaviour. The reversibility of the cyclic voltammograms obtained in acetonitrile is in contrast with that reported for MV^2+^ in aqueous solutions in which radical dimerisation occurs after the first one‐electron reduction.^45^ The second reduction is not as well behaved because the neutral product is less soluble and highly susceptible to reaction with oxygen. We therefore focus on the kinetics of the first reduction.

**Table 1:** Formal potentials for the first one‐electron ($E_{a}^{o}$ ) and second one‐electron ($E_{b}^{o}$ ) reduction of MV^2+^ and compounds **C1**–**C5** measured by cyclic voltammetry. The scan rate was 0.1 V s^−1^ and the potentials are reported against an Ag/Ag^+^ (10 mm) reference in MeCN with 0.1 m TBAPF_6_ as electrolyte.

	$E_{a}^{o}$ [mV]	$E_{b}^{o}$ [mV]
MV^2+^	−759	−1179
**C1**	−684	−1119
**C2**	−914	−1197
**C3**	−1034	−1278
**C4**	−1030	−1312
**C5**	−1070	−1314

**Figure 2:**
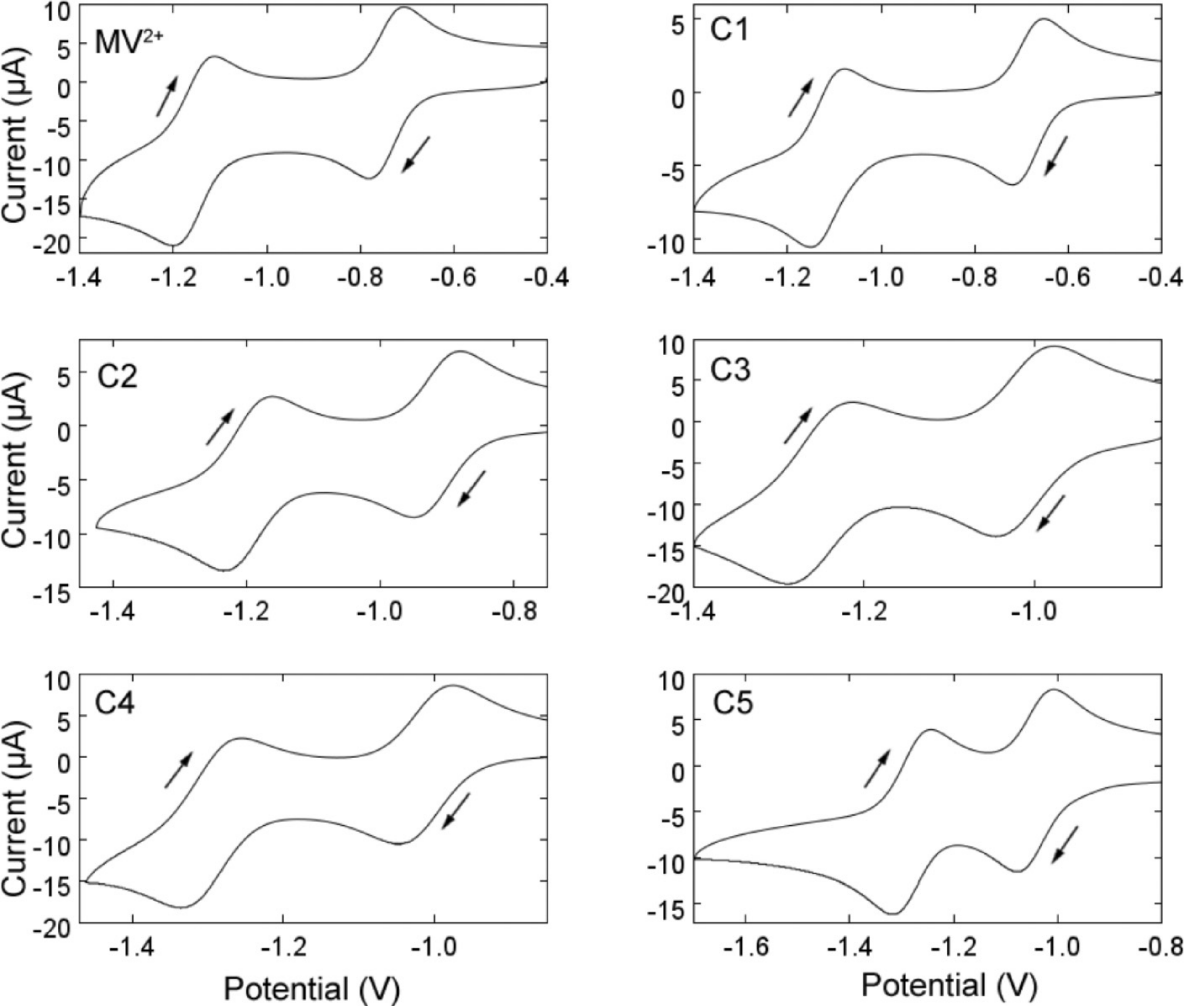
Cyclic voltammograms of MV^2+^ and derivatives **C1**–**C5** obtained at a 1 mm radius Pt disc electrode in 0.1 m TBAPF_6_/MeCN solution with an analyte concentration of 1 mm and a scan rate of 0.1 V s^−1^. The initial scan direction was negative. Potentials are reported with respect to an Ag/Ag^+^ (10 mm, MeCN) reference electrode.

A more detailed study of the first reduction was made by conducting a combination of cyclic voltammetry at Pt macroelectrodes (1 mm radius, 0.1 V s^−1^) (Figure 2) and steady‐state voltammetry at Pt microelectrodes (25 μm radius). These measurements allow us to test the usual assumption that the diffusion coefficients of the oxidised and reduced forms of the redox couple are approximately the same. The ratio of the diffusion coefficients are thus determined according to Equation (1) where Δ*E*_1/2_ is the difference between the formal potential estimated from the time‐dependent CV experiment and the halfwave potential *E*_1/2_ from the steady‐state experiment. All other symbols have their standard electrochemical meanings. The measured Δ*E*_1/2_ values are given in Table 2 and are all only a few millivolts in magnitude. The diffusion coefficients of the dication and the reduced monocation were therefore equal for each couple within experimental uncertainty. This leads to a simplification of the kinetic analysis of the viologen redox couples.

(1) $$\Delta E_{1/\;2}=\frac{RT}{2nF}ln\frac{D_{R}}{D_{O}}$$

**Table 2:** Differences between the formal potentials measured by time‐dependent (CV at 1 mm radius Pt disc, 0.1 V s^−1^) and steady‐state (25 μm radius Pt disc microelectrode) voltammetry for the one‐electron reduction of MV^2+^ and compounds **C1**–**C5** in MeCN with 0.1 m TBAPF_6_ as electrolyte.

	Δ*E*_1/2_ [mV]
MV^2+^	1
**C1**	3
**C2**	1
**C3**	4
**C4**	3
**C5**	2

**Figure 3:**
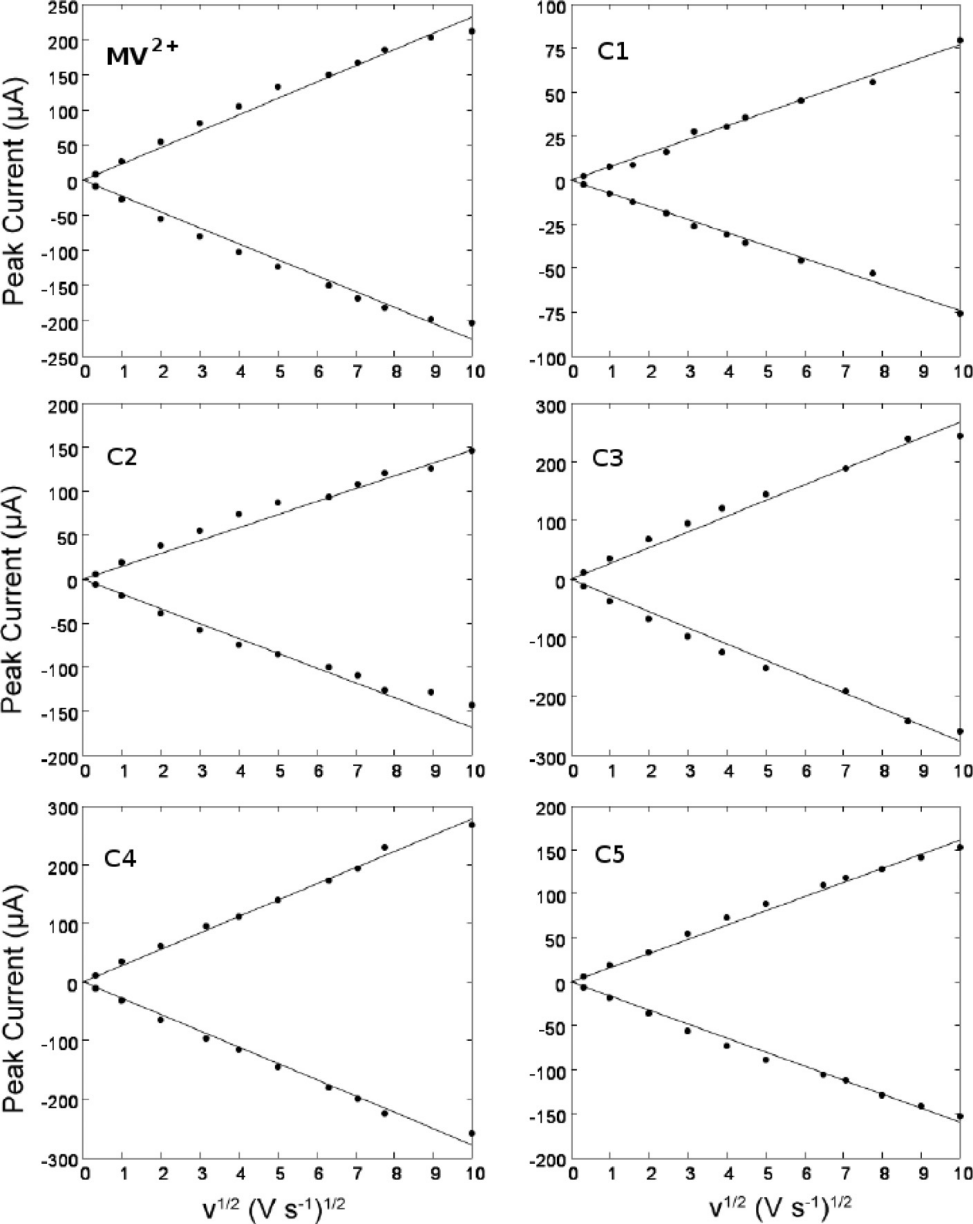
Peak currents for the forward (cathodic) and reverse (anodic) wave for the first one‐electron reduction of MV^2+^ and **C1**–**C5** show the expected linear dependence on the square root of scan rate. Data collected by cyclic voltammetry at a 1 mm radius Pt disc in 0.1 m TBAPF_6_/MeCN electrolyte and 1 mm analyte concentration. No background subtraction has been applied.

Previous studies in aqueous solution have found evidence that methyl viologen adsorbs on the surface of the working electrode, complicating the analysis of kinetic data.^43^ However, in MeCN we find that the peak currents for both the forward (cathodic) and reverse (anodic) processes were directly proportional to the square root of the scan rate over the range 0.1 to 100 V s^−1^, which indicates that adsorption of neither the dication nor the radical cation occurred to any significant extent in MeCN.^48^ This holds for MV^2+^ and all the derivatives **C1**–**C5** (Figure 3).

As well as adsorption, electrode fouling by products of reactions of the radical cation or neutral species is a possible complication in kinetic studies. To test the stability of the voltammetry, cyclic voltammograms of MV^2+^ were taken once every 15 seconds over a period of 30 minutes at a 1 mm radius Pt electrode. The formal potential did not change between voltammograms; this shows that oxygen did not enter the system during this time, because oxygen shifts the peak potential to more positive values by reacting with the radical cations. We can therefore rule out any influence of oxygen on the kinetic measurements. We did however find that the peak current for both the forward and reverse waves decreased and the separation between the peaks increased over the course of the 30 minute test. This behaviour is consistent with fouling of the Pt electrode by decomposition products of the methyl viologen radical.

Overall, these findings suggest that the first one‐electron redox reactions of these molecules are well suited to kinetic study in acetonitrile. However, the slow electrode fouling effect that was observed suggests that more accurate data can be obtained from techniques that do not require the electrode potential to be poised for long periods at potentials at which the radical cation forms.

### 2.2 Differential Capacitance of Pt and Bi Electrodes in Acetonitrile

To interpret the electron‐transfer kinetics at Bi and Pt electrodes, some knowledge of their interfacial energetics must be obtained; in particular, the potential distribution must be known so that the Frumkin correction can be made. We therefore carried out a study of the differential capacitance of Pt and Bi in MeCN/TBAPF_6_ electrolyte. Electrochemical impedance spectra (EIS) of Pt and Bi electrodes in electrolytes comprising 0.1 m TBAPF_6_ in anhydrous acetonitrile were found to be well represented by simple series RQ circuits (defined below) and analysed as such to determine the differential capacitance of the systems. Measurements were carried out by using a Pt wire quasi‐reference, which was periodically compared against an Ag/Ag^+^ reference electrode with a Keithley 6430 high impedance voltmeter. All potentials are reported with respect to Ag/0.01 m Ag^+^. The low impedance of the Pt wire made it possible to collect reliable EIS data at higher frequencies than were accessible with the Ag/Ag^+^ reference and the drift in the Pt QRE potential was about 6.5 mV during the potential scan.

Both the Pt and Bi electrodes show constant phase element behaviour in background electrolyte. The impedance spectra were fitted to a series “RQ” circuit where *Q* represents a constant‐phase element and the exponent *γ* lies between 0 and 1 [Eq. (2)].

(2) $$Z=R+\frac{1}{{\left(i\omega \right)}^{\gamma }Q}$$

When *γ*=1 the constant‐phase element is an ordinary capacitor. This is approximately the case for Bi/MeCN where we observed *γ*>0.96 over the whole range of potentials studied. However, as is typical at solid electrodes, we observed values significantly less than 1 for Pt/MeCN, i.e. *γ*≃0.87. In such a case the “true capacitance” of the electrode is not *Q*. An effective capacitance can be defined as -1ωIm(Z) , however this is clearly frequency‐dependent when *γ*≠1. In principle, the true differential capacitance is defined by the thermodynamic relation $c=\frac{\partial \sigma }{\partial E}$ where *σ* is the charge density, but the effective capacitance of the constant‐phase element has no such DC limit. Either a more sophisticated model circuit should be used to fit the data^49^ or an appropriate frequency must be chosen. We are interested in the differential capacitance primarily to make the Frumkin correction for our heterogeneous electron‐transfer rate data obtained by fast CV; therefore, we propose that the appropriate angular frequency is of the order of $2\pi f=\omega =\frac{V}{\left(E{{}^{\circ }}^{'}-E_{\mathrm{pzc}}\right)}$ where *V* is the scan rate in V s^−1^. This choice can be justified because the double layer does not attain the charge determined by the time‐independent capacitance $c=\frac{\partial \sigma }{\partial E}$ in the fast CV experiment. It also matches the upper limit of frequencies for which we were able to obtain rate data from impedance spectroscopy.

The differential capacitance‐potential data for both Pt/MeCN and Bi/MeCN showed the expected dip, which allowed identification of the potentials of zero charge (*E_pzc_*) as −0.60 V (Bi) and −0.43 V (Pt). We modelled the data by using three capacitors in series [Eq. (3)]. The differential capacitance of the Helmholtz layer, *c*_H_, and the diffuse layer, *c*_D_, were calculated according to the standard expressions of the Gouy–Chapman–Stern model^48^ and the third contribution, *c*_SM_, arises from the possibility of a significant potential drop within the electrode (*E*_SM_). In principle, *c*_SM_ may be important for a semi‐metal such as Bi and was calculated according to Equation (4) where *ρ*(*E*_F_) is the density of states at the Fermi‐level and *θ*_0_ is given by Equation (5).^50^, ^51^ The value of *ϵ*_r_ for Bi was taken to be 99.6 as obtained from IR reflectivity measurements.^52^ We assume that, near the Fermi energy, the local density of states varies linearly with energy $\rho \left(E\right)=\rho \left(E_{F}\right)+\beta \left(E-E_{F}\right)$ and *β* is a constant that we float in the regression analysis below. Note that in the case of Pt, *c*_SM_ is too large to have any significant effect on the value of *c*.

(3) $$c^{-1}=c_{\mathrm{SM}}^{-1}+c_{H}^{-1}+c_{D}^{-1}$$

(4) $$c_{\mathrm{SM}}=\frac{e}{2}\sqrt{\epsilon_{r}\epsilon_{0}}\frac{\alpha k_{B}T\theta_{0}^{2}+2\rho \left(E_{F}\right)\theta_{0}}{\sqrt{\frac{1}{3}\alpha k_{B}T\theta_{0}^{3}+\rho \left(E_{F}\right)\theta_{0}^{2}}}$$

(5) $$\theta_{0}=\frac{F(E_{\mathrm{SM}})}{RT}=\frac{e}{k_{B}T}\left(E_{\mathrm{SM}}\right)$$

A change in the electrode potential of *δφ* results in changes in the potential difference across the Helmholtz layer, the diffuse layer and the solid of *δφ*_H_, *δφ*_D_, and *δφ*_SM_ respectively [Eq. (6)]. The ratio of the potential change across the diffuse layer to the total potential change is given in terms of the differential capacitances by Equation (7). Analogous equations exist for the potential change across the Helmholtz layer and inside the solid electrode. The differential capacitance at the potential of zero charge was calculated directly from Equation (3), Equation (4), standard Gouy–Chapman theory and the relevant parameters (OHP, ionic strength, *ρ*(*E*_F_)). The differential capacitance over a range of potentials was then obtained by numerical integration of Equation (6) and Equation (7) by using an explicit finite difference technique with potential increments of 1 mV.

(6) $$\delta \phi =\delta \phi_{H}+\delta \phi_{D}+\delta \phi_{\mathrm{SM}}$$

(7) $$\frac{\delta \phi_{D}}{\delta \phi }=\frac{1}{1+\frac{c_{D}}{c_{\mathrm{SM}}}+\frac{c_{D}}{c_{H}}}$$

**Figure 4:**
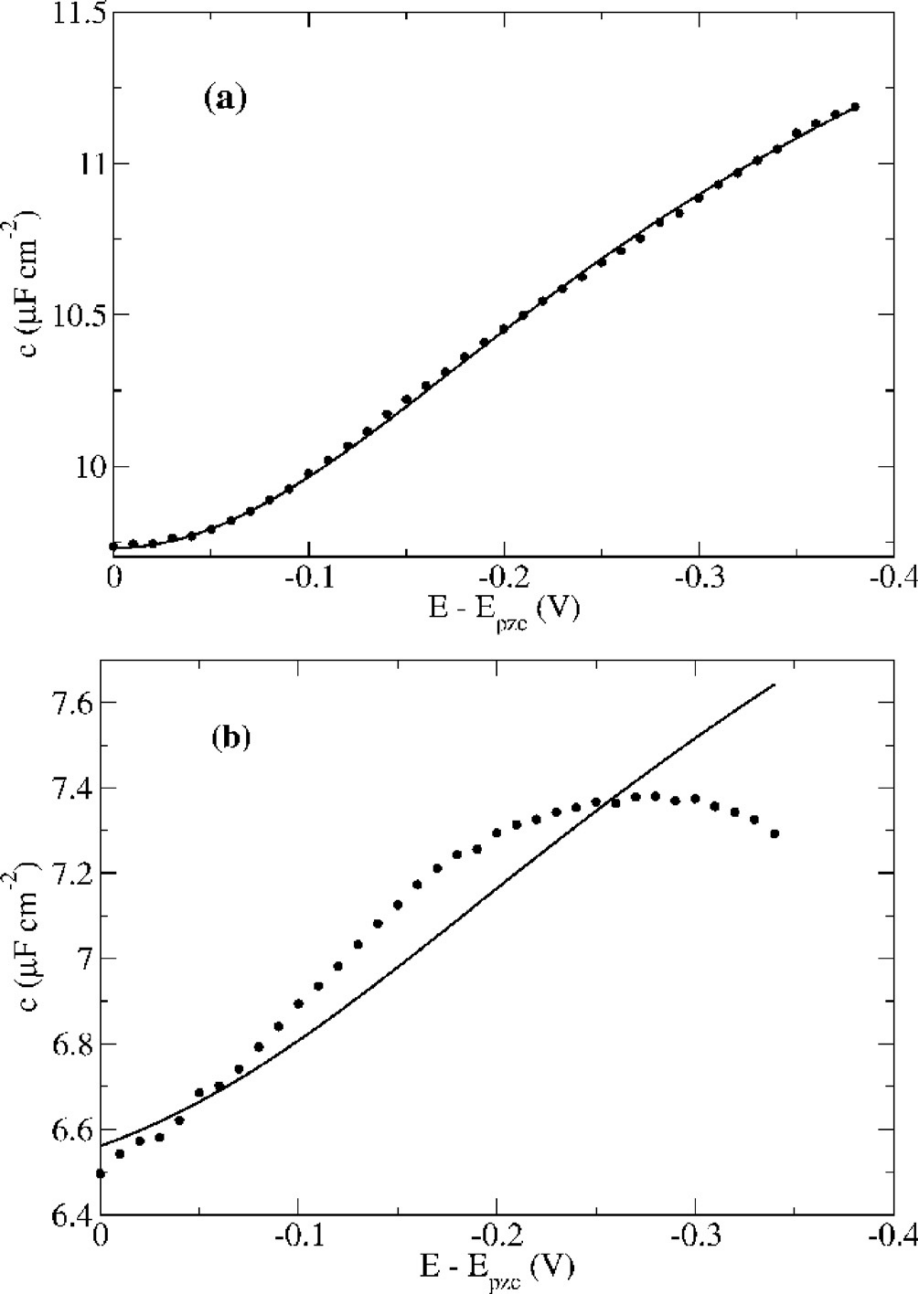
Differential capacitance values measured at a) Pt, *E_pzc_*=−0.43 V and b) Bi, *E_pzc_*=−0.60 V, in a 0.1M TBAPF_6_/MeCN electrolyte. Data were measured over the frequency range 1–50 kHz; the Bi data were nearly independent of frequency, but for Pt constant‐phase behaviour was observed with an exponent *γ*=0.87. The Pt data plotted are the effective differential capacitance for *f*≅32 kHz as discussed in the text. The solid line represents the regression model defined by Equations (3)–(7).

Differential capacitance values, for a frequency of 32 kHz (*ω*=2×10^5^ rads^−1^) at a 1 mm radius Pt electrode, measured over a range of potentials negative of the potential of zero charge (*E_pzc_*) and relevant to the electron‐transfer measurements, are plotted in Figure 4 a. Also shown is the least squares fit of our regression model, defined by Equations (3)–(7), to the experimental data. The density of states for Pt was taken as 1.2×10^23^ cm^−3^ eV^−1^.^53^ As expected, this large value makes the calculated *c*_SM_ for Pt so high that the contribution of *c*_SM_ to the overall differential capacitance of the Pt/MeCN,TBAPF_6_ interface is negligible. The solid line was calculated according to the regression model detailed above. The bulk value of relative permittivity for MeCN was taken as 37.5 and the value for the Helmholtz layer as 3.0. The distance to the outer Helmholtz plane was allowed to float to obtain the best fit (2.1 Å). The ionic strength (which enters via the diffuse layer capacitance) was also floated to account for possible TBA^+^:PF_6_^−^ ion pairing within the solution.

Differential capacitance curves have been reported previously for Bi in aqueous,^25^ ethanol^26^, ^49^ and acetonitrile electrolytes.^22^, ^27^ Measurements at one of our 0.5 mm radius Bi electrodes are also shown in Figure 4. A comparison of the Pt and Bi data shows that although the differential capacitance of the Bi/MeCN interface is smaller, the difference is only about 30–40 %. Indeed, if the bulk Fermi‐level density of states for Bi is estimated by using the measured bulk carrier density of 3×10^17^ cm^−3[54, 55]^ and the same value of Helmholtz layer thickness as at Pt (2.1 Å) is used to compute the differential capacitance at the PZC, a value of 0.6 μF cm^−2^ is obtained. The agreement between the model and the experimental *c*−*E* data for Bi/MeCN is not as good as for Pt/MeCN and a more sophisticated analysis accounting for effects such as specific adsorption would be required to obtain quantitative agreement.^27^ However, it is clear that the measurements are inconsistent with the bulk Fermi‐level density of states for Bi. To obtain the rough agreement between the data and the model shown in Figure 4 b, a density of states of 9×10^20^ cm^−3^ eV^−1^ was required. There is precedent for such a large difference between the DOS in the bulk and at the Bi surface for (111), (110) and (100) surfaces.^56^ In addition, scanning tunnelling spectroscopy experiments have estimated the surface density of states of the (100) Bi surface to be between 2.8×10^21^ and 2.8×10^22^ cm^−3^ eV^−1^,^57^ the lower limit is in rough agreement with our estimate from differential capacitance measurements.

### 2.3 Heterogeneous Electron‐Transfer Kinetics of MV^2+^ and C1–C5

#### 2.3.1 Fast‐Scan Voltammetry

**Figure 5:**
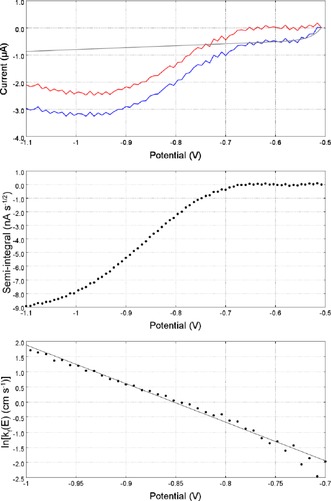
Top: A cyclic voltammogram recorded at scan rate 20 kV s^−1^ for MV^2+^ (10 mm) in MeCN with TBAPF_6_ (0.2 m) at a 5 μm radius platinum disc shown in blue. A fitted background is shown in grey and the background subtracted data shown in red. Middle: A semi‐integral plot of the background subtracted voltammogram. Bottom: A plot of ln[*k*_f_ (cm s^−1^)] as a function of potential; the potential range is constrained to the linear region which occurs after *E°′*.

**Figure 6:**
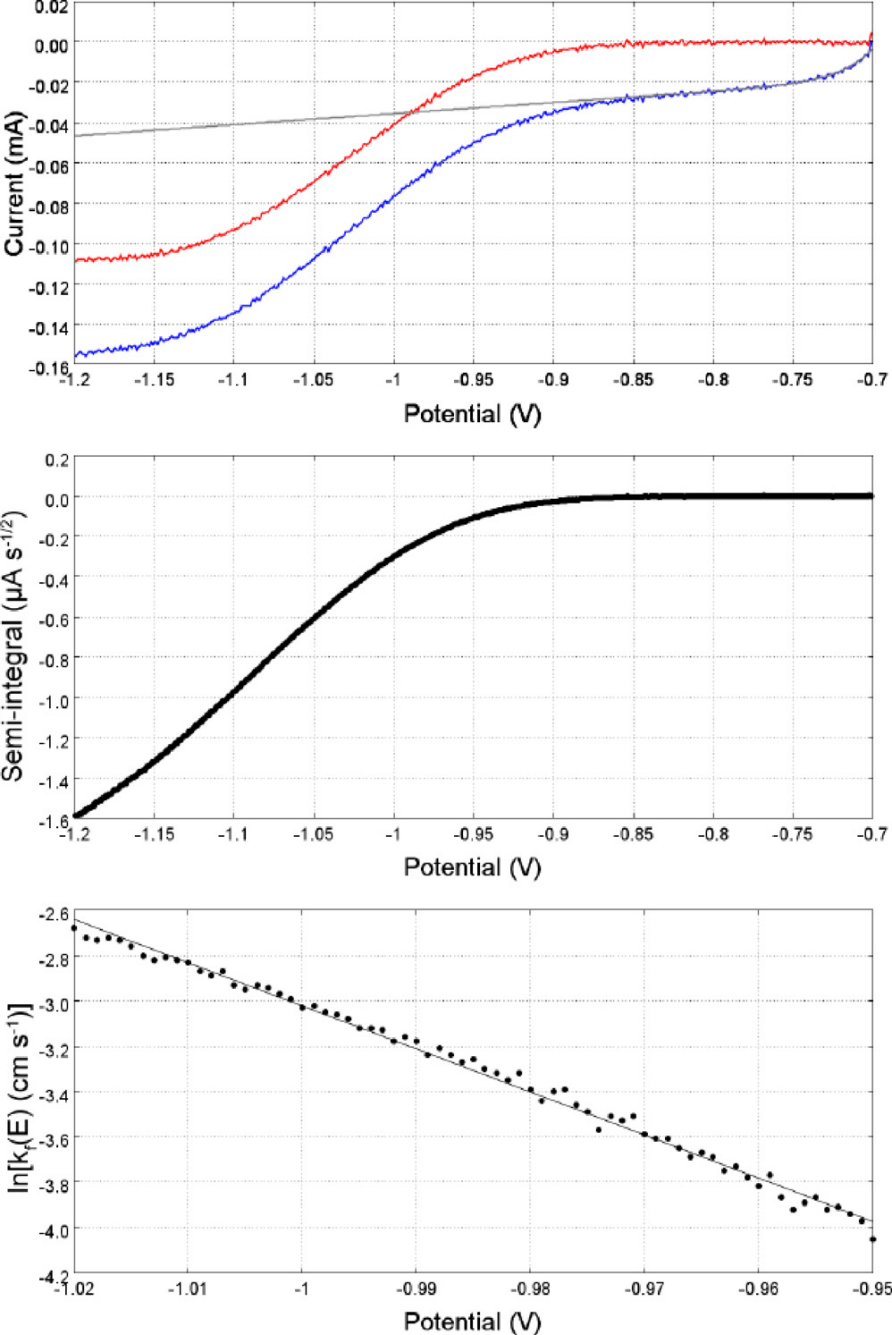
Top: A cyclic voltammogram recorded at scan rate 1 kV s^−1^ for C4 (10 mm) in MeCN with TBAPF_6_ (0.2 m) at a 0.5 mm radius bismuth disc shown in blue. A fitted background is shown in grey and the background subtracted data shown in red. Middle: A semi‐integral plot of the background subtracted voltammogram. Bottom: A plot of ln[*k*_f_ (cm s^−1^)] as a function of potential with the potentials constrained to the linear region occurring after *E°′*.

Cyclic voltammograms taken at a 5 μm radius Pt microelectode, with a scan rate of 20 kV s^−1^ were used to determine the standard rate constant, *k* ^0^, and transfer coefficient, *α*, for the one‐electron reduction of MV^2+^ and **C1**–**C5** at Pt/MeCN. In each case, 50 voltammograms were co‐added and averaged to improve the signal to noise ratio after ensuring that no clear differences existed between individual voltammograms, beside noise. A two‐electrode cell, with the Ag/Ag^+^ electrode acting as both reference and counter, was used within a grounded Faraday cage. Solutions of analyte at 10 mm concentration in 0.2 m TBAPF_6_/MeCN were used. The values of *k* ^0^ and *α* were determined by using the methods detailed by Saveant and Tessier.^47^ Fast‐scan voltammograms for **C1** and ln(*k*_f_) for its one‐electron transfer are shown as a function of potential in Figure 5. These experiments were then repeated at a 0.5 mm radius Bi electrode at a scan rate of 1 kV s^−1^. The corresponding voltammograms and ln(*k*_f_) plots obtained from Bi are shown in Figure 6.

Each voltammogram contained some background current that had a significant effect upon the measured *α* value, and a smaller effect on the measured *k* ^0^. Equation (8) was fitted to the region of the voltammogram in which no faradaic current was observed using a standard least squares method. *E* and *E*_0_ represent the potential at time *t* and the initial potential; *m*_1_, *m*_2_, *I*_0_ and *c* are parameters adjusted in the fitting procedure. The second term models the transient at the beginning of the scan. After estimating the background current in this manner, it was subtracted from the entire voltammogram and the kinetic analysis was applied.

(8) $$I_{\mathrm{background}}=\left(m_{1}\left(E-E_{0}\right)-I_{0}\right)+m_{2}\exp\left(c(E-E_{0})\right)$$

In all cases, nearly linear plots of ln(*k*_f_) against potential were obtained. This and the agreement with rates measured by using EIS below, suggests that our simple background subtraction is sufficient to treat the data. More sophisticated analyses would take migration effects and the coupling of solution resistance and electrode capacitance into account, but the major electric field effect is the Frumkin correction, which is treated later. The standard rate constants and transfer coefficients obtained at Pt and Bi are given in Table 3 and Table 4.

**Table 3:** Standard rate constants (*k* ^0^) and transfer coefficients (*α*) determined for the one‐electron reduction of MV^2+^ and **C1**–**C5** at Pt from 20 kV s^−1^ voltammograms. All measurements were performed with an analyte concentration of 10 mm in MeCN and 0.2 m TBAPF_6_ as electrolyte.

Pt data	*k* ^0^ [cm s^−1^]	*α*
MV^2+^	1.4×10^−1^	0.34
**C1**	1.1×10^−1^	0.34
**C2**	1.1×10^−1^	0.63
**C3**	1.7×10^−1^	0.45
**C4**	1.6×10^−1^	0.40
**C5**	2.8×10^−1^	0.50

**Table 4:** Standard rate constants (*k* ^0^) and transfer coefficients (*α*) determined for the one‐electron reduction of MV^2+^ and of **C1**–**C5** at Bi from 1 kV s^‐1^ voltammograms. All measurements were performed with an analyte concentration of 10 mm in MeCN and 0.2 m TBAPF_6_ as electrolyte.

Bi data	*k* ^0^ [cm s^−1^]	*α*
MV^2+^	1.8×10^−2^	0.44
**C1**	1.3×10^−2^	0.52
**C2**	2.4×10^−2^	0.73
**C3**	4.7×10^−2^	0.48
**C4**	2.8×10^−2^	0.48
**C5**	1.5×10^−2^	0.47

This method of determining *k* ^0^ does not require that the electrode be held at a negative potential for any significant amount of time; the time taken for each scan is between approximately 50 μs and 1 ms. We observed no decrease in the current measured between runs; this is consistent with the electrode remaining unfouled by reaction products.

#### 2.3.2 Electrochemical Impedance Spectroscopy

Electrochemical impedance spectroscopy (EIS) was used to obtain kinetic data for methyl viologen and the derivatives **C1**–**C5** (concentrations=1 mm) in 0.1 m TBAPF_6_/MeCN electrolyte at a 5 μm radius Pt disc. The measurements employed a frequency range of about 1 Hz to 30 kHz, with the applied dc potential set to the appropriate formal potential to determine the charge‐transfer resistance (*R*_ct_). For the purpose of analysis, the system was treated as a Randles equivalent circuit.^48^ Uncompensated resistance (*R*_Ω_) was found to be negligible because in all the Nyquist plots the data tended to the origin at high frequencies. The real component of the impedance can be described by Equation (9) and the imaginary component by Equation (10), where *W* is given by Equation (11).

(9) $$Z{}^{'}=R_{\Omega }+\frac{R_{\mathrm{ct}}+\sigma \omega^{-1/2}}{W^{2}+\omega^{2}C_{d}^{2}{\left(R_{\mathrm{ct}}+\sigma \omega^{-1/2}\right)}^{2}}$$

(10) $$-Z{}^{''}=\frac{\omega C_{d}{\left(R_{\mathrm{ct}}+\sigma \omega^{-1/2}\right)}^{2}+\sigma \omega^{-1/2}W}{W^{2}+\omega^{2}C_{d}^{2}{\left(R_{\mathrm{ct}}+\sigma \omega^{-1/2}\right)}^{2}}$$

(11) $$W=C_{d}\sigma \omega^{1/2}+1$$

Before we apply Equations (9)–(11) to the data, we justify the use of equations appropriate to semi‐infinite linear diffusion to analysis of data from a microelectrode. The faradaic impedance for a reversible reaction at a microhemisphere, *Z*, is described by Equation (12), where *Ω* is given by Equation (13) and *i*(*E*) is the reversible steady‐state voltammogram.

(12) $$Z^{-1}=\left(1+\sqrt{i\Omega }\right){\left(\frac{\partial i}{\partial E}\right)}_{\mathrm{rev},\mathrm{SS}}$$

(13) $$\Omega =\frac{a^{2}\omega }{D}$$

The Randles circuit for a planar electrode has no finite DC impedance, i.e. it corresponds to the case where $1+\sqrt{i\Omega }≅\sqrt{i\Omega }$ in Equation (12). For this to be true with 5 % error, we must have frequencies high enough so that Equation (14) is satisfied.

(14) $$\frac{\left|\sqrt{i\Omega }\right|}{\left|1+\sqrt{i\Omega }\right|}>0.95$$

Thus, from Equation (15), for *D*=10^−5^ cm^2^ s^−1^ and *a=*5×10^−4^ cm:

(15) $$f=\frac{\omega }{2\pi }>83\;\mathrm{Hz}$$

This analysis ignores possible corrections due to the non‐uniformity of the current distribution at the microdisc, but for frequencies in the kHz region, the approximation is sufficient for the precision of our experimental data.

**Figure 7:**
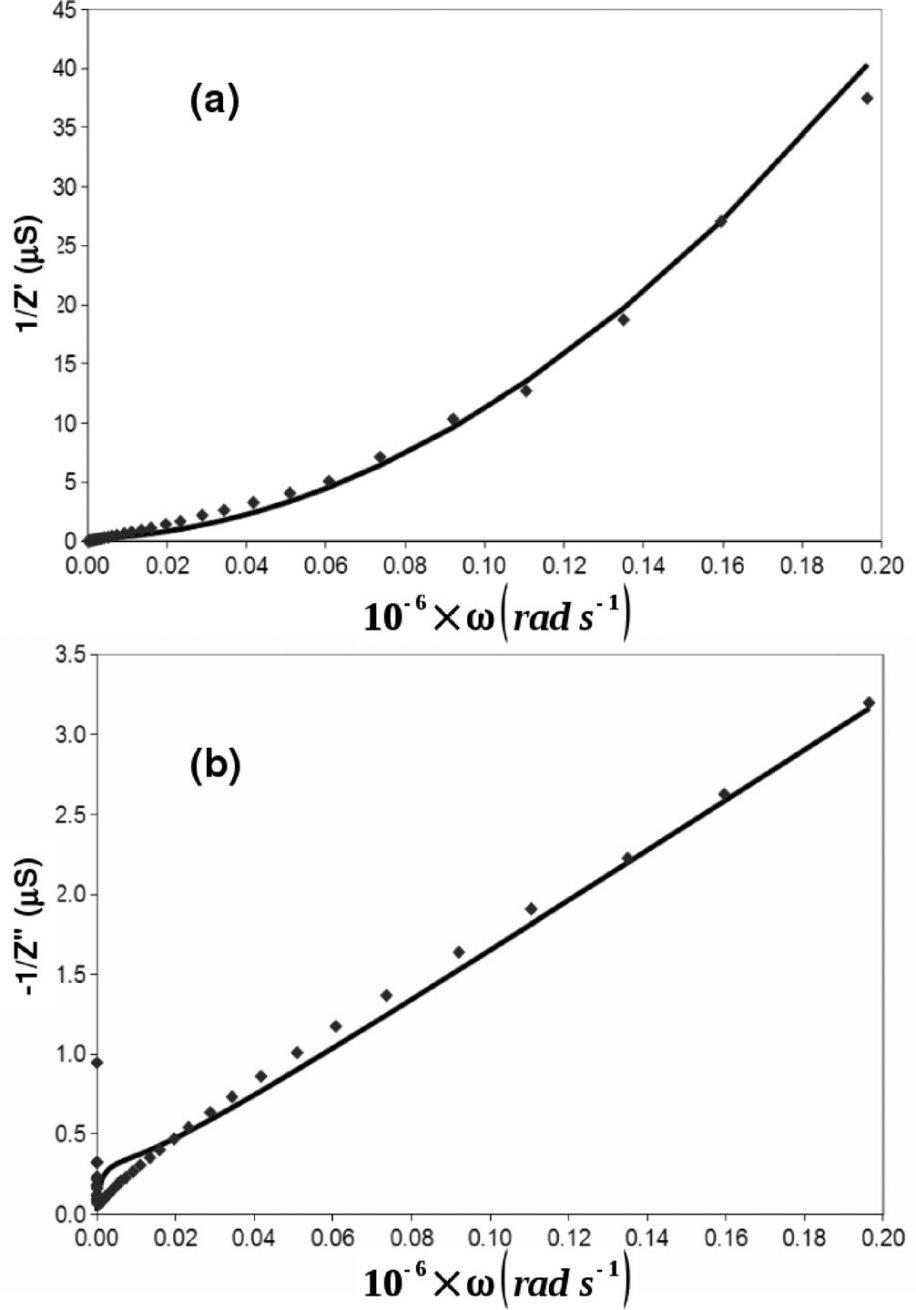
Impedance data recorded in MeCN with 0.1 m TBAPF_6_ and 1 mm C2 at a 5 μm radius Pt microdisc. a) 1/*Z*′, where *Z*′ is the real part of the impedance; b) −1/*Z*′′ where *Z*′′ is the imaginary part of the impedance. Reciprocal impedance values are plotted to place emphasis upon the high‐frequency data.

At high frequencies another simplification is possible. The Warburg impedance becomes insignificant and the impedance of the cell can be described by Equation (16) and Equation (17). Plots of 1/*Z*′ and −1/*Z*′′ against *ω* were used to emphasise the high‐frequency data and extract values of charge‐transfer resistance and *k* ^0^ by using the standard expressions.^48^ High‐frequency data was collected by using fast Fourier transform techniques and data collection was fast enough (several seconds) that any adsorption on to the electrode was unlikely to be significant given our observations on absorption in slow‐scan cyclic voltammetry experiments mentioned above. The values of *k* ^0^ determined for the compounds are listed in Table 5 and Figure 7 shows typical impedance data collected for C2. In general, there is rough agreement with the data obtained by fast‐scan voltammetry at Pt—the trends in values are similar and the rates are of the same order of magnitude.

(16) $$Z{}^{'}=\frac{R_{\mathrm{ct}}}{1+\omega^{2}C_{d}^{2}R_{\mathrm{ct}}^{2}}$$

(17) $$-Z{}^{''}=\frac{\omega C_{d}R_{\mathrm{ct}}^{2}}{1+\omega^{2}C_{d}^{2}R_{\mathrm{ct}}^{2}}$$

**Table 5:** Values of *k* ^0^ found for MV^2+^ and compounds **C1**–**C5** using electrochemical impedance spectroscopy at a 5 μm radius Pt microdisc.

Pt data	*k* ^0^ [cm s^−1^]
MV^2+^	2.(4)×10^−1^
**C1**	1.(9)×10^−1^
**C2**	2.(8)×10^−1^
**C3**	3.(2)×10^−1^
**C4**	4.(8)×10^−^
**C5**	9.(0)×10^−1^

Attempts to extend the EIS measurements to obtain spectra over a range of dc potentials around the formal potential and to obtain values of transfer coefficient, *α* were unsuccessful. Slow changes in the parameters were observed and unreliable values obtained. We suspect these effects are a result of the adsorption/fouling behaviour of the reduction products, which is inevitable when the electrode is poised at negative potentials. The short time‐scale (roughly 50 μs) of the fast CV experiments allowed data collection to be completed before electrode fouling could significantly impact the results. Attempts to obtain EIS data for Bi electrodes were entirely unsuccessful because of time‐dependent behaviour due to fouling. This may be explained by the smaller rate of diffusion of products away from the surface of the larger Bi electrodes.

#### 2.3.3 Steady‐State Voltammetry using SECM

Electrochemical microscopy was used in an attempt to independently confirm the kinetic parameters obtained through EIS and fast CV studies. These experiments were performed at a 5 μm radius Pt disc SECM tip at an unbiased Pt substrate.

A tip approach to within 0.5 μm of the Pt substrate was accomplished before a steady‐state cyclic voltammogram was obtained. Attempts were then made to fit this voltammogram to the theory for a uniformly accessible, steady‐state voltammogram.^58^ However, the voltammograms were found to be reversible and, in the case of C4, not retraceable. A likely cause is slow electrode fouling observed in the impedance experiments. Although steady‐state voltammetry has advantages for fast kinetic studies,^59^ this illustrates a case in which fast CV is superior because it avoids slow electrode fouling processes caused by the products of the electron‐transfer reaction.

### 2.4 A Comparison of Electron‐Transfer Rates at Pt and Bi

The standard rate constant observed at a semi‐metallic electrode surface ($k_{\mathrm{sm}}^{0}$ ) may be quantitatively related to that observed at a metallic electrode ($k_{m}^{0}$ ), with knowledge of the density of states, atomic densities and reorganisation energies at each interface as given by Equation (18).^6^ *D*, *d*, *λ*, *q* and *E*_PZC_ represent the Fermi‐level density of states (in units of cm^−3^ eV^−1^), the atomic density and the reorganisation energy, the charge on the electrode, and the potential of zero charge, respectively. This approximation assumes the nonadiabatic limit applies, $D\left(-eE{}^{\circ }{}^{'}\right)≅D\left(-eE_{\mathrm{pzc}}\right)$ , that the electronic coupling per state and the distance dependences are the same for the metal and the semi‐metal.

(18) $$k_{\mathrm{sm}}^{0}=k_{m}^{0}\frac{D_{\mathrm{sm}}\left(-eE_{\mathrm{pzc}}\right)}{D_{m}}{\left(\frac{d_{\mathrm{sm}}}{d_{m}}\right)}^{\frac{2}{3}}{\left(\frac{\lambda_{m}}{\lambda_{\mathrm{sm}}}\right)}^{\frac{1}{2}}\exp\left(\frac{\lambda_{m}-\lambda_{\mathrm{sm}}}{4k_{B}T}\right)$$

The measured relative permittivity of Bi is large (99.6^52^) and therefore we expect that electrostatic contributions from image forces at the Bi and Pt interfaces are similar. In that case, Equation (18) predicts that density of states effects will be the major factor in the difference between rates at metallic and semi‐metallic electrodes.

Before a meaningful comparison of rates at Pt and Bi/MeCN interfaces can be made, the data must be corrected to account for the effect of the electrode charge at the formal potential of each dication on the standard rate constants. This requires knowledge of the potential at the outer Helmholtz plane (*φ*_2_) at the formal potential of the couple. The necessary *φ*_2_ values were estimated from differential capacitance data at the appropriate electrolyte concentration using the least squares fit of the regression model given by Equations (3)–(7). The Frumkin‐corrected rate constant, $k_{\mathrm{FC}}^{0}$ , can then be determined by use of Equation (19) where *z* is the charge on the analyte.^48^

(19) $$k_{\mathrm{FC}}^{0}=k^{0}\exp\left(-\frac{\left(\alpha -z\right)F\phi_{2}}{RT}\right)$$

**Figure 8:**
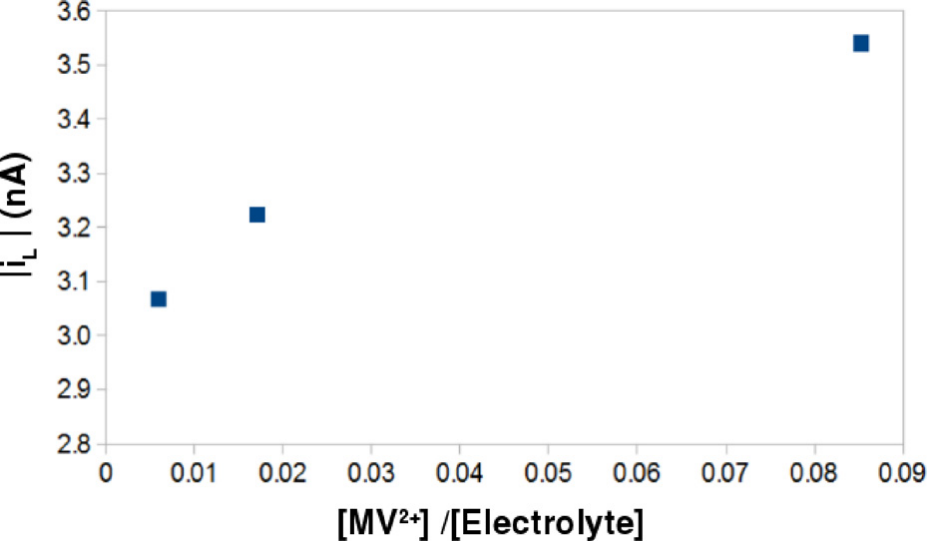
Limiting current of steady‐state voltammograms at a 5 μm radius Pt microdisc electrode against analyte/electrolyte concentration ratio. The solutions contained 1 mm MV^2+^ and varying concentrations of TBAPF_6_ in MeCN. The transport‐limited current increases as supporting electrolyte concentration decreases.

In principle, ion‐pairing might affect the appropriate value of *z* to use with Equation (19). However, the charge‐diffuse nature of the viologens appears to make this effect negligible in the electrolyte used. Evidence for this comes from the variation of steady‐state transport‐limited currents at 5 μm Pt microelectrodes with the concentration of electrolyte [TBAPF_6_] (Figure 8).

The increase in current with decrease in electrolyte concentration (ca. 15 %) shown in Figure 8 is consistent with a migration effect arising from screening of the electrode charge at high electrolyte concentrations. The magnitude of the effect is similar to previous reports for MV^2+^/dimethyl ether and MV^2+^/TBAClO_4_ based on quantitative theoretical models and *z*= +2.^60^, ^61^ Additional support for the choice *z*=+2 comes from the observation that if we apply the Frumkin correction for *z=*1 or *z=*0 we do not observe any correlation between rates at Pt and Bi.

**Figure 9:**
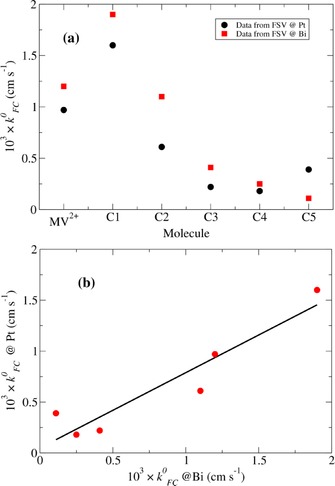
The Frumkin‐corrected standard rate constants, $k_{\mathrm{FC}}^{0}$ , determined at Pt and Bi for each molecule are shown in (a). Plot (b) shows the Pt rate constants plotted against the Bi rate constants, with a line drawn according to the linear regression of the Pt data on the Bi data.

The values of the Frumkin‐corrected standard rate constants $k_{\mathrm{FC}}^{0}$ are given in Table 6 and plotted in Figure 9. The atomic densities of 6.6×10^22^ cm^−3^ for Pt and 2.8×10^22^ cm^−3^ for Bi^62^ are within a factor of 3. It is therefore clear that, if the ET reaction is non‐adiabatic, the Fermi‐level density of states at the surface of Bi must be very different than in the bulk (where the carrier density is of the order of 10^17^ cm^−3^ 54, 55) and much closer to that for Pt, which is 1.2×10^23^ cm^−3^ eV^−1^.^53^ The rate data suggest a much larger value than we obtain from the differential capacitance data (9×10^20^ cm^−3^ eV^−1^). Possible explanations include 1) the ET reaction is adiabatic;^4^ 2) estimates of density of states from the differential capacitance data become less precise as the surface becomes metal‐like because the potential drop within the solid becomes small and 3) we are making an assumption of equal reorganisation energies at Pt and Bi, which is hard to test directly. Finally, we note that the Frumkin correction is large even in 0.2 m TBAPF_6_/MeCN and that assessments of the DOS‐dependence of ET rates for highly charged ions require great care. We have also not corrected for dynamic diffuse layer effects^20^ or the distance dependence of electron transfer.^63^

**Table 6:** Frumkin‐corrected standard rate constants at Pt and Bi obtained by fast‐scan voltammetry (FSV).

	Bi $k_{\mathrm{FC}}^{0}$ [cm s^−1^]	Pt $k_{\mathrm{FC}}^{0}$ [cm s^−1^]
**MV^2+^**	1.2×10^−3^	9.7×10^−4^
**C1**	1.9×10^−3^	1.6×10^−3^
**C2**	1.1×10^−3^	6.1×10^−4^
**C3**	4.1×10^−4^	2.2×10^−4^
**C4**	2.5×10^−4^	1.8×10^−4^
**C5**	1.1×10^−4^	3.9×10^−4^

### 2.5 The Effect of Viologen Structure on the Heterogeneous Electron‐Transfer Kinetics

The molecular volume and inter‐ring torsion angles of the molecules are collected in Table 7. Given the similarities in the molecules studied here, we expect that the reorganisation energy, *λ*, will be the main source of variation in $k_{\mathrm{FC}}^{0}$ between molecules. Furthermore, we might predict that, with the exception of C5, the solvation spheres of the molecules will be similar enough to allow the outer‐sphere reorganisation energy *λ*_o_ to be considered roughly equal in each case ($\lambda_{o}\propto \mathrm{radius}\propto \frac{1}{\sqrt[{3}]{V}}$ ). Comparison of the geometries of the dication and monocation forms of each molecule, as modelled at the B3LYP/6‐31G(d) level of theory, suggests that the major inner sphere reorganisation is related to the decrease in the torsion angle between the two pyridine rings.^30^

**Table 7:** Molecular volume and torsion angle between the pyridine rings for MV^2+^ and the derivatives **C1**–**C5**. The molecular volumes were obtained from geometry‐optimised structures at the B3LYP/6‐31G(d) level of theory (Spartan04, Wavefunction Inc., CA, USA) and the torsion angles are from similar calculations, but with inclusion of solvent effects by the polarised continuum model, reported previously.^30^

Molecule	Volume [Å^3^]	Dication torsion angle [°]
MV^2+^	215	43.0
**C1**	238	35.7
**C2**	256	62.3
**C3**	274	51.3
**C4**	292	56.1
**C5**	393	54.1

**Figure 10:**
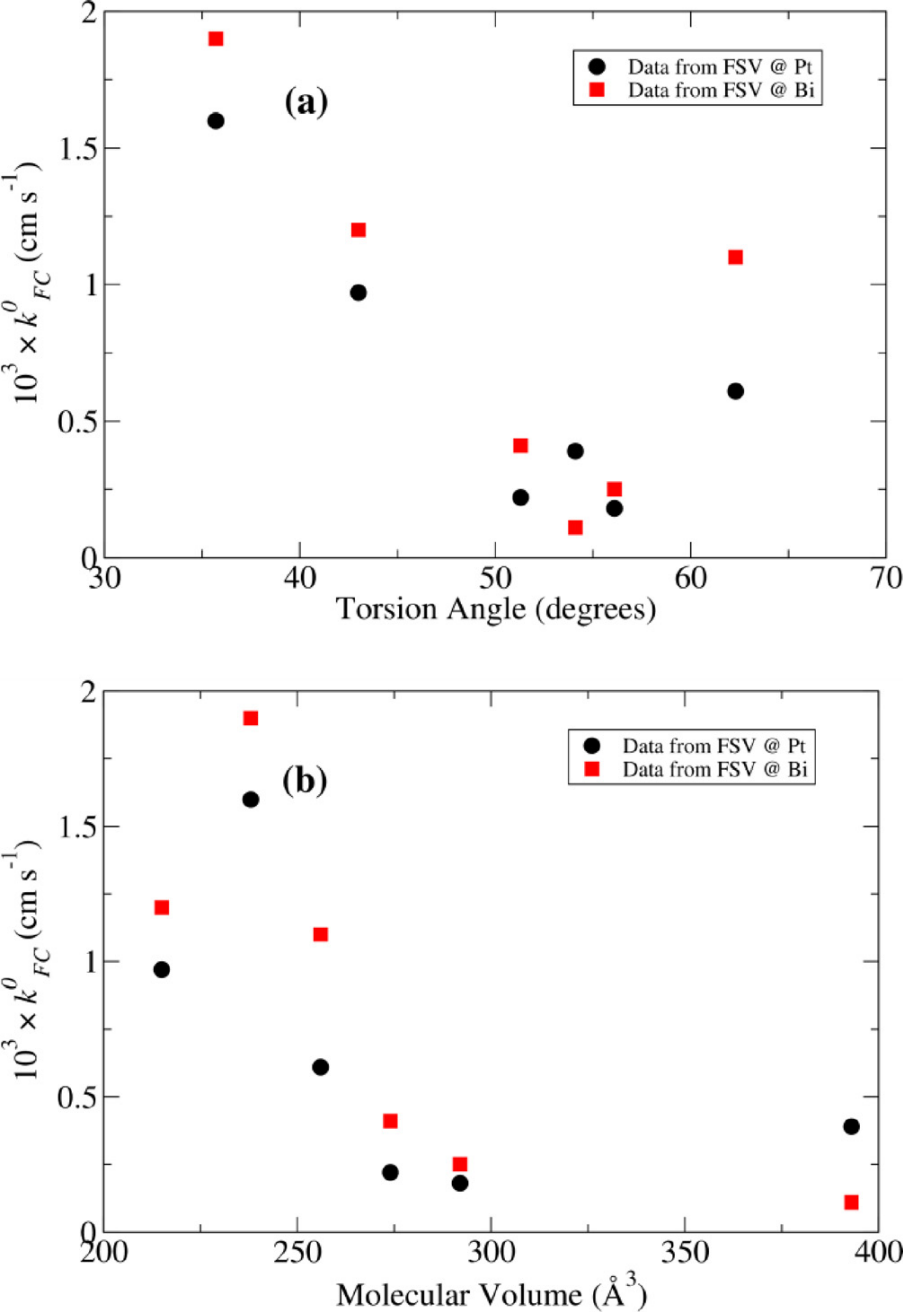
The Frumkin‐corrected standard rate constants, $k_{\mathrm{FC}}^{0}$ , determined at Pt and Bi for each molecule plotted against a) the inter‐ring torsion angle and b) the molecular volume.

The measured $k_{\mathrm{FC}}^{0}$ values are plotted against calculated torsion angles in Figure 10 a and a reasonable negative correlation (a rank correlation coefficient=−0.60) was observed for the Pt FSV data. Only C2, with the largest torsion angle, is clearly not in the trend. Computational models at the B3LYP/6‐31G(d) level indicate that the LUMO of each dication is located almost entirely on the pyridine rings. Therefore, an effect of the side chain may be to introduce an orientation requirement that must be met before electron transfer can occur. In general we might expect that the rate will decrease with molecular volume if there are preferred orientations for electron transfer. There is a partial downward trend in $k_{\mathrm{FC}}^{0}$ with molecular volume as apparent in Figure 10 b, provided one discounts the *k* ^0^ value measured for MV^2+^ (the molecule with the lowest molecular volume and least hindered rotation about the inter‐ring bond). Finally, it is worth noting that if the outer sphere reorganisation energy were the main difference between these molecules, the rate ought instead to increase slightly with molecular volume.

## 3 Conclusions

A combination of differential capacitance and heterogeneous electron‐transfer measurements at Bi and Pt electrodes indicates that the electrochemical behaviour of Bi is not compatible with the bulk density of states at the Fermi level. These measurements show that the differential capacitance at the Bi/MeCN interface is only about 30–40 % smaller than at Pt/MeCN. In addition, the electron‐transfer rates for a series of viologen derivatives at Bi/MeCN are comparable to those at Pt/MeCN; this is suggestive of adiabatic behaviour, but it should be noted that a surface density of states of a similar order of magnitude to that for Pt/MeCN would also explain the data. The viologen derivatives also showed an interesting inverse correlation between the standard rate constant and the inter‐ring torsion angle of the viologen dication. We suggest that the reorganisation energy is larger for derivatives that have large torsion angles in the dication form, because viologen cation radicals tend towards planarity as a result of partial double bond character in the inter‐ring carbon–carbon bond.
